# Supplementary material for: Molecular characterization and spatiotemporal expression of prohormone convertase 2 in the Pacific abalone, Haliotis discus hannai
Source: PLoS One. 2020 Apr 9;15(4):e0231353. doi: 10.1371/journal.pone.0231353 (PMC7144994; doi:10.1371/journal.pone.0231353)
Supplement: S1 Raw Images — (PDF) [file pone.0231353.s001.pdf]

Gel image

For molecular cloning of PC2 gene, we did reverse transcription PCR for searching partial sequence. The following Fig.1 is the uncropped gel picture of PC2.

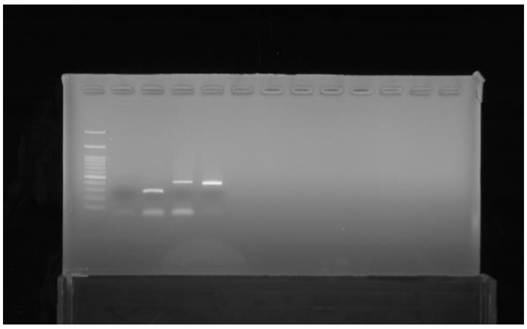

Fig. 1 Gel picture of PC2 for searching partial sequence

After getting the partial sequence we did 3′- RACE and 5′RACE PCR. The following Fig.2 and Fig. 3 is the uncropped gel picture of 3′- RACE and 5′RACE PCR, respectively.

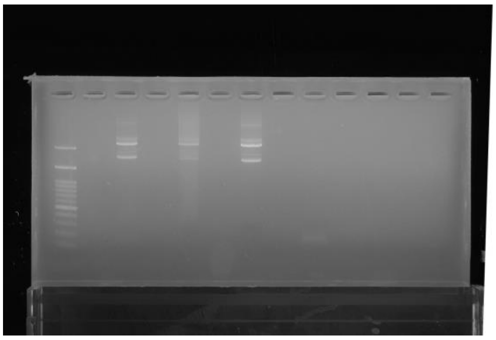

Fig. 2 3′-RACE PCR gel picture

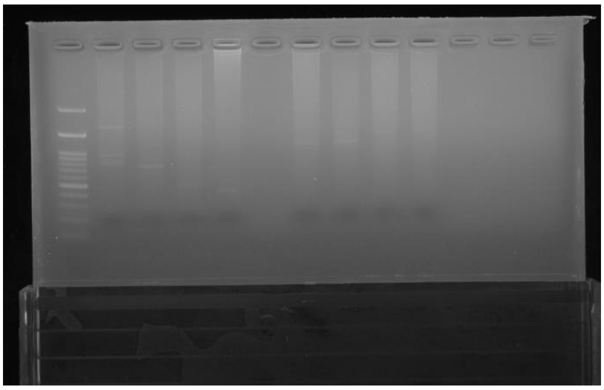

Fig. 3 5′-RACE PCR gel picture

After getting the full-length sequence of PC2, we did Semi-quantitative RT-PCR. The following Fig. 4 and Fig. 5 indicate the semi-quantitative RT PCR of Hdh PC2 and RPL-5 in Pacific abalone tissues, respectively. After checking the gel picture, we conducted qPCR

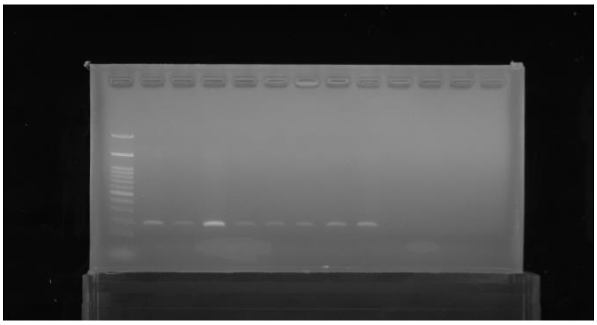

Fig. 4 Semi-quantitative RT-PCR expression of Hdh PC2 in Pacific abalone tissues

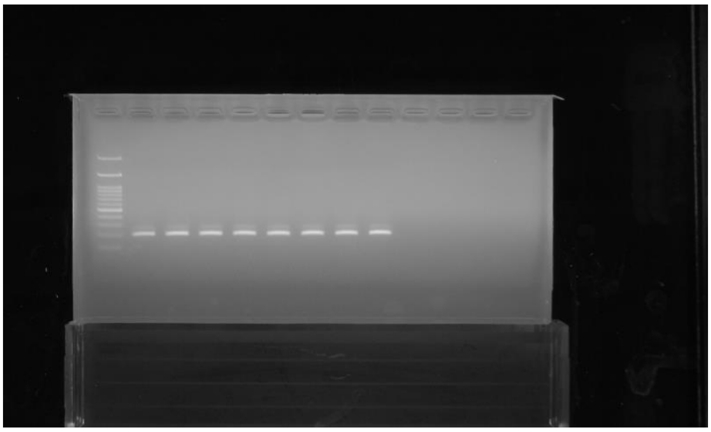

Fig. 5 Semi-quantitative RT-PCR expression of RPL-5 in Pacific abalone tissues
