# Supplementary material for: Molecular characterization and spatiotemporal expression of prohormone convertase 2 in the Pacific abalone, Haliotis discus hannai
Source: PLoS One. 2020 Apr 9;15(4):e0231353. doi: 10.1371/journal.pone.0231353 (PMC7144994; doi:10.1371/journal.pone.0231353)
Supplement: Attachment — Submitted filename: Response to reviewers.docx [file pone.0231353.s002.docx]

Reviewer #1: The manuscript by Sharker and colleagues describes the identification and functional characterization of a prohormone convertase from the pleuropedal ganglion of the abalone Haliotis discus hannai. Although most of the analyses have been carried out quite efficiently, there are several points of this manuscript that require attention. I feel like the text could be improved by removing the unnecessary section about the structural modeling (it is unclear why this was performed by the authors since the results have not been discussed) and removing the parts concerning semi-quantitative PCR (which was just used as a preliminary analysis for qRT-PCR). Please find my detailed report attached below.
Line 143: This section is largely deficient in terms of the data provided. First, how were such sequences selected? Were all the sequences available in public databases used? Why were two different phylogenetic inference mathods (NJ and ME) used? In figure 3, the authors only reported the NJ tree, so it is needless to include the mention of the ME tree here.

**Response:** We thank the reviewer for pointing this out and we agree with the reviewer. Therefore, we have added such information. To construct a phylogenetic tree, PC2 protein sequences from invertebrates and vertebrates were retrieved from the NCBI database using the BLASTP algorithm. This sentence is incorporated into lines151-152. Based on your valuable suggestion, I omitted the minimum evolution method from the line number 154.

Lines 149-150: too little information has been provided. It is important to specify which were the top scoring templates used for modeling (i.e. are other similar 3D structure available in the PDB database?).

**Response:** We thank the reviewer for pointing this out and we agree with the reviewer. Therefore, we have revised it accordingly and omitted from the abstract (line number 38-40) materials and methods (line number 155-160), and result (line number 266-275)section.

Lines 171-172: “The melting temperature was 95°C for 10 s, 65°C for 1 min, and 97°C for 1 s as a default setting.”. This sentence seems out of place, as the cycling conditions have been described above. What are the authors referring to? I guess this sentence was supposed to describe the melting curve analysis, but its description is completely wrong.

**Response:** We thank the reviewer for pointing this out and we agree with the reviewer. Therefore we revised it and omitted this sentence from the line number 182.

Line 217: “This is the Fig 1 title.” -> ???

**Response:** Based on the journal format, I incorporated the Fig. legend.

Line 226: “In silico analysis indicated that the deduced protein might be localized in the plasma membrane.” -> This is not very clear, considering that the following sentences do not describe any transmembrane domain. Which in silico analyses specifically indicate a plasma membrane localization? Based on literature data, what would be the expected subcellular localization of PC2? Is it common to find PC2 associated with the plasma membrane?

**Response:** We thank the reviewer for pointing this out and we agree with the reviewer. Therefore, we have added such information. In silico analysis (protcomp, <http://linux1>.softberry.com/berry.phtml) indicated that the sub-cellular localization of this deduced protein is in the plasma membrane. This sentence is incorporated into lines 233-234.

Line 256: “monophyletc outgroup”? What do the authors mean by this? This should be simply defined as “outgroup”.

**Response:** We thank the reviewer for pointing this out and we agree with the reviewer. Therefore, we revised it accordingly as shown in line number 261.

Lines 265-266: “with a C-score between 2 and 4” -> NO. The C-score ranges from -4 and 2 and a given model is only characterized by a unique c-score, not by a range.
Lines 267-270: several critical information are missing here. Reporting proQ and ERRAT scores is quite useless if the authors do not report which structural templates were used for modeling. More in general, it looks like the structural modeling part had little sense in this manuscript, as it was not discussed in any meaningful way. My impression is that the authors were not very expert in the use of I-TASSER (which is a very easy-to-use online tool) and that their ability to discuss the outcome of the modeling process was very limited. Since the 3D structure of PC2 is not discussed in any way in this manuscript and the authors obviously have little idea about what they are doing here, I suggest that this part should be entirely removed, since it is pointless for the aims and scopes of this manuscript. The discussion found in lines 342-344 is very poor and uninformative. What was the reason to include structural modeling data in this manuscript is this is actually completely irrelevant for the discussion of the findings? The semi-quantitative PCR analysis can be removed from the text. Obviously, this was just performed as a preliminary analysis to set-up the q-PCR analysis, which is a much superior method. Please remove this part (from the materials and methods and from the results section) and only leave the qPCR results.

**Response:** Based on your valuable suggestion, I omitted the 3D structure of PC2 and semi quantitative PCR from materials and methods, results, and discussion section. I also incorporated the following sentence in the line number 289-291.

qPCR assay was performed to investigate the mRNA expression profile of Hdh PC2 in neural ganglia (cerebral ganglia, branchial ganglia, pleuropedal ganglia), digestive gland, gonad (testis, ovary), gill, and mantle using gene-specific and ribosomal protein L-5 (RPL-5) primers.

Line 348: this should be expanded. What types of hormones are usually produced by this organ? Note that most of the readers of PlosONE are not familiar with abalone anatomy.

**Response:** We thank the reviewer for pointing this out and we agree with the reviewer. Please see the following sentences

Neuroendocrine hormone and their receptor such as GnRH (Kim et al., 2017), GnRH receptor (Sharker et al., unpublished), serotonin receptor (Sharker et al., 2020) and also steroid enzymes 17β HSD-11 (Sharker et al., unpublished) are synthesized from pleuropedal ganglion. PC2 (Neuroendocrine/prohormone convertase) seems to be involved in the posttranslational process of neuroendocrine hormone.

Line 546: CLUSTALW -> wasn’ ClustalOmega used (see materials and methods)

**Response:** We thank the reviewer for pointing this out and we agree with the reviewer. Therefore we have inserted it as shown in line number 548.
