# Supplementary material for: Molecular characterization and spatiotemporal expression of prohormone convertase 2 in the Pacific abalone, Haliotis discus hannai
Source: PLoS One. 2020 Apr 9;15(4):e0231353. doi: 10.1371/journal.pone.0231353 (PMC7144994; doi:10.1371/journal.pone.0231353)
Supplement: Attachment — Submitted filename: Response to reviewers.docx [file pone.0231353.s003.docx]

Reviewer #1: Thank you for providing a revised version of this manuscript. I feel like the manuscript has been significantly improved with the removal of the 3D modeling part and a better definition of some key passages of the main text.
There are however some important points that still require attention.
1) The authors mention the use of Softberry to predict the subcellular localization of this protein, but provided a link to an outdated version of this portal. The authors should state that the tools used for this prediction is ProtComp 9.0 and provide the appropriate reference. Moreover, it appears that the subcellular localization of the protein is not the plasma membrane, but the Golgi apparatus. I have retrieved the sequence myself using the accession ID provided by the authors and verified that the predicted subcellular localization is Golgi. This would make much more sense than having a transmembrane localization, as proteolytic processing often happens in the Golgi network (see for example https://doi.org/10.1074/jbc.M507193200. I would urge the authors to correct this result and add necessary literature references to works that report the expected subcellular localization of similar proteases.

**Response:** We thank the reviewer for pointing this out and we agree with the reviewer. Therefore, we have added such information. *In silico* analysis (protcomp, http://www.softberry.com/berry.phtml) indicated that the sub-cellular localization of this deduced protein is in the membrane bound golgi network. This sentence is incorporated into lines 216-217. We have also added ‘The predicted protein might be localized in the membrane bound golgi network which is in agreement with the results of previous studies [6]’ in the line number 306-307.

2) The authors updated their phylogenetic tree and mentioned that the sequences added were detected through BLASTp. Which selection criteria were used? Any specific threshold of primary sequence homology? The authors should explain whether other paralogous genes were present and how these were excluded.

**Response:** Thank you very much for your valuable comments. We did not consider any specific threshold for selecting sequence homology. Based on your valuable suggestions, we included paralogous genes of PCs and reconstructed the phylogenetic tree. We have added following information in the line numbers 241-243 and inserted the reconstructed phylogenetic tree figure (Fig. 3)

‘The constructed phylogenetic tree revealed several distinct clades. Hdh PC2 is contained in the gastropod PC2 clade and is more closely related to Has PC2’.
